# Can you see what I am talking about? Human speech triggers referential expectation in four-month-old infants

**DOI:** 10.1038/srep13594

**Published:** 2015-09-01

**Authors:** Hanna Marno, Teresa Farroni, Yamil Vidal Dos Santos, Milad Ekramnia, Marina Nespor, Jacques Mehler

**Affiliations:** 1 Language, Cognition and Development Lab, SISSA, Via Bonomea 265, Trieste, 34136, Italy; 2 Psychology Department, University of Padova, Via 8 Febbraio 1848, 2, 35122 Padova, Italy; 3 Language, Cognition and Development Lab, SISSA, Via Bonomea 265, Trieste, 34136, Italy; 4 Language, Cognition and Development Lab, SISSA, Via Bonomea 265, Trieste, 34136, Italy; 5 Language, Cognition and Development Lab, SISSA, Via Bonomea 265, Trieste, 34136, Italy; 6 Language, Cognition and Development Lab, SISSA, Via Bonomea 265, Trieste, 34136, Italy

## Abstract

Infants’ sensitivity to selectively attend to human speech and to process it in a unique way has been widely reported in the past. However, in order to successfully acquire language, one should also understand that speech is a referential, and that words can stand for other entities in the world. While there has been some evidence showing that young infants can make inferences about the communicative intentions of a speaker, whether they would also appreciate the direct relationship between a specific word and its referent, is still unknown. In the present study we tested four-month-old infants to see whether they would expect to find a referent when they hear human speech. Our results showed that compared to other auditory stimuli or to silence, when infants were listening to speech they were more prepared to find some visual referents of the words, as signalled by their faster orienting towards the visual objects. Hence, our study is the first to report evidence that infants at a very young age already understand the referential relationship between auditory words and physical objects, thus show a precursor in appreciating the symbolic nature of language, even if they do not understand yet the meanings of words.

Human language is a special auditory stimulus for which infants show a unique sensitivity, compared to any other types of auditory stimuli. Various studies found that newborns are not only able to distinguish languages they never heard before based on their rhythmical characteristics^1^,^2^,^3^,^4^, but they can also detect acoustic cues that signal word boundaries^5^, discriminate words based on their patterns of lexical stress^6^ and distinguish content words from function words by detecting their different acoustic characteristics^7^. Moreover, they can also recognize words with the same vowels after a 2 min delay^8^. In fact, infants are more sensitive to the statistical and prosodic patterns of language than adults, which provides an explanation of why acquiring a second language is more difficult in adulthood than during infancy^9^. In addition to this unique sensitivity to the characteristics of language, infants also show a particular preference for language, compared to other auditory stimuli. For example, infants at the age of 2-months, and even newborns prefer to listen to speech compared to non-speech stimuli, even if the non-speech stimuli retain many of the spectral and temporal properties of the speech signal^10^,^11^.

Thus, there is growing evidence that infants are born with a unique interest and sensitivity to process human language. But what are exactly the grounds and the scopes of this privileged status of speech? Why do infants orient themselves towards speech stimuli with a distinguished attention? And why are they tuned to process it in a unique way, compared to any other auditory stimuli? One may argue that due to its human-specific nature, speech can be perceived as a “trait” of our conspecifics, and as such, it may help social bonding or enable to establish group membership. In this way, similarly to other social traits (such as race or gender, e.g.^12^,^13^,^14^), speech can be especially interesting to infants, because it may provide some guidance in the surrounding social world. Indeed, numerous studies showed that infants not only prefer their native language^15^, but that during social learning, when they observe a model, sharing the same language with this model can also influence their attitudes and choices (e.g.^16^,^17^,^18^).

However, besides this social saliency of language, it might be that infants are receptive towards speech because they also understand that speech can communicate *about* something. More specifically, they might understand that speech can convey information about the surrounding world and that words can refer to specific entities. Indeed, without this understanding, they would have great difficulty to accept relations between objects and their labels, and thus language acquisition would become impossible. Furthermore, without understanding the referential relations between words and objects, learning about the surrounding world would become extremely difficult. Hence, we propose that in order to successfully acquire language, conceiving of speech as a meaningful referential symbolic system must be a predisposition of infants already present at a very early age. Furthermore, as a guiding principle for learning, speech should also effectively bias infants to seek potential referents of the words they hear.

With the aim to study infants’ knowledge about the function of speech, numerous studies have investigated infants’ understanding of the role of speech in communication. These studies showed that infants at the age of 12-months^9^,^10^,^11^,^12^,^13^,^14^,^15^,^16^,^17^,^18^,^19^,^20^ and even 6-month-olds^21^ can understand that speech can communicate about unobservable intentions of the speaker. Furthermore, a recent study also gave evidence that those infants, who show greater interest to listening to speech sounds, compared to non-speech, will score higher on expressive vocabulary measures at the age of 18 months^22^. Thus, attentiveness towards speech can be also a precursor of later language development.

However, in order to successfully acquire a language and the meaning of concepts, more than accepting the communicative nature of speech and being attentive to it, one should also understand the direct referential relationship between words and the entities they stand for. In the past there has been some evidence showing that young infants already accept some relations between verbal labels and physical objects. For example, providing verbal labels can facilitate object categorization of 3-4-month-old infants, and this effect disappears when they listen to sequences of tones^23^,^24^. At the age of 12-months, they can also individuate objects when they hear two labels, but not when they hear two tones or perceive two emotional expressions^25^. Furthermore, at the same age, they can also associate words but not communicative sounds (e.g. ‘mmm’ or ‘aaah’) or consonantal sounds to novel objects^26^. Listening to verbal labels can also modulate object perception, as a study by Gliga *et al.*^27^ showed: 12-month-old infants displayed enhanced gamma-band oscillatory activity over the visual cortex when they saw objects along with verbal labels. Furthermore, 12- and 19-month-old infants can associate words with complex objects, rather than with their salient parts^28^. Moreover, words can guide their attention towards the commonalities among a set of named objects, and it was suggested that this guidance in turn builds up a symbolic reference to form a set of stable “word-to world” mappings^29^.

These findings suggest that during development, some recognition of object-label relations may precede language acquisition. But the evidence that infants would have a basic expectation about the symbolic nature of verbal labels and that they would know that words can directly stand for other entities (e.g. objects) in the world is still missing from the literature. In the present study we address this question: as a precursor of understanding the referential relation between spoken words and real objects in the world, do infants from a very young age expect to find the referents of the words they hear? If it is true that the early sensitivity to the characteristics of language demonstrated by human infants also indicates an expectation about the referentiality of language, then we predict that infants should look for potential referents in their environment only when they hear somebody talking but not when the auditory signal lacks the characteristics of human speech (ie. backward speech or no speech at all).

## Experiment 1

**Figure 1:**
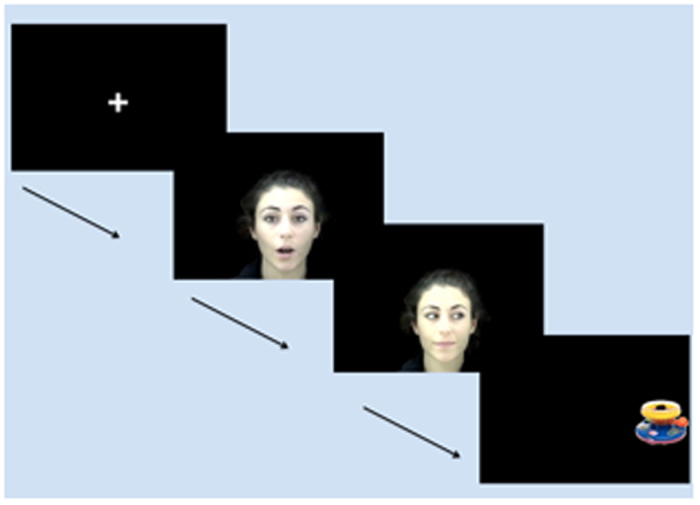
Design of Experiment 1. Each movie started with a fixation cross in the middle of the center of the screen, then a female face appeared and vocalized or silently moved her lips. Following this, she looked to the right or to the left direction of the screen. Then the face appeared and an object appeared, in a congruent direction with the eye-gaze.

Thirty, 4-month-old infants were tested using a looking time latency paradigm, and were shown videos of a female face, who was either talking in a normal way (Normal Speech Condition), or backward (Backward Speech Condition), or she was silently moving her lips (No Speech Condition), while she was looking at the infant. We selected backward speech because even though its auditory characteristics are very similar to those of normal speech, there is evidence that neither infants, nor adults process backward speech similarly to natural languages^4^,^30^,^31^. In each trial, the movie ended with an averted eye-gaze of the speaker either to the left, or to the right side of the screen. Next, the face disappeared from the display and immediately after an object appeared in a direction congruent with the gaze (Fig. 1. and see examples of videos in the Supplementary Material). The latency of infants’ orienting to the objects was measured as the dependent variable.

## Results of Experiment 1

**Figure 2:**
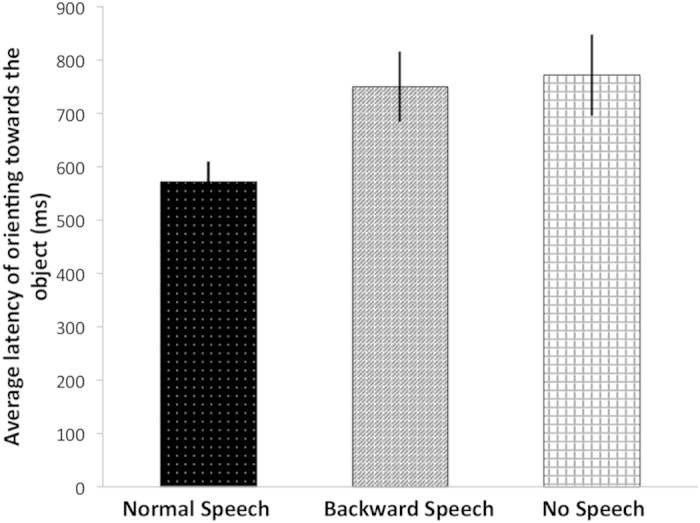
Results of Experiment 1. Latency of fixation towards the visual object. Bars represent the average latency of thirty infants’ orienting towards the visual object in the three auditory conditions. Error bars indicate SEM.

We predicted that if infants have a referential expectation about language, they would expect to find the referent and therefore orient faster to the object in the Normal Speech than in the Backward Speech or the No Speech condition. This prediction was confirmed by our data (Fig. 2). A 3 × 1 Repeated Measures ANOVA analyzing the latency of infants’ orienting to the objects in the three conditions showed a main effect of condition [*F*(2, 58) = 4.403, *p* = 0.017, *η*^*2*^ = 0.132]. Bonferroni-corrected post-hoc pairwise tests revealed that infants looked significantly faster at the object in the Normal Speech condition than in the Backward [*t*(29) = −2.935, *p* = 0.006] and the No Speech condition [*t*(29) = −2.640, *p* = 0.013]. Between the Backward and the No Speech condition there was no significant difference [*t*(29) = −0.262, *p* = 0.795]. These results confirm that when infants hear normal language, they are faster in finding the referent of speech compared to when they hear non-linguistic stimuli or when they only see the silent movements of the lips. Thus, the speech can facilitate infants’ search for the referent of speech at the age of 4 months.

However, since in the stimuli we always used an object-directed gaze of the speaker, congruent with the direction of appearance of the object, it remains unclear whether without this object-directed eye-gaze we would get the same effect, or the combination of speech and an object-directed gaze of the speaker is needed. To answer this question, we designed a second experiment, where in addition to the averted gaze trials, we also included trials where the speaker was gazing at the infant during the entire movies. We predicted that in case an object-directed gaze is needed to elicit referential expectation, this would result a significant difference in infants’ looking time latency between the averted object-directed gaze conditions, and the new infant-directed gaze conditions. Additionally, in case we find that an averted (thus possibly object-directed) eye-gaze is necessary to evoke the searching behaviour for the referent, it would be interesting to estimate the power of this factor. To this end, we decided to use *incongruent* averted gaze trials as well, i.e. conditions where the direction of the eye-gaze was averted from the infant, but incongruent with the appearance of the object. The extent to which these trials slow down the searching behaviour of infants indicates the strength of the effect of eye-gaze in eliciting referential expectation.

## Experiment 2

In order to clarify these questions, we collected N = 30 participants’ data in a second experiment with 3 × 3 factors, referring to Language (Normal Speech, Backward Speech and No Speech), and Gaze (Congruent Object-Directed, Incongruent Object-Directed and Infant-Directed). An additional change of the stimuli was that with the aim to decrease the rejection rate of our subjects, we slightly increased the size of the face, in order that infants would find the task more attractive and that they would stay attentive during the entire experiment. The task was the same as in Experiment 1. While infants watched the stimuli, their looking time latency was measured in each trial towards the objects.

## Results of Experiment 2

We analyzed the data in a 3 × 3 analysis of variance (ANOVA) with Language (Normal Speech vs. Backward Speech vs. No Speech) and Gaze (Object-Directed Congruent vs. Object-Directed Incongruent vs. Infant-Directed). There was a main effect of Language [*F*(2, 58) = 4.037, *p* = 0.023, *η*^*2*^ = 0.122] and a strong interaction of Language and Gazing [*F*(4, 116) = 7.166, *p* = 0.0001, *η*^*2*^ = 0.198]. Following this, we analyzed the effect of Language independently for each Gaze conditions. This analysis revealed a strong significant difference between the three levels of Language conditions in the Congruent Object-Directed Gaze condition [*F*(2, 58) = 11.844, *p* = 0.0001, *η*^*2*^ = 0.290]. In the Incongruent Object-Directed and in the Infant Directed Gaze conditions, however, we found no significant differences. We also performed 2 × 3 analysis of variance with Language and Gaze to contrast the Language conditions separately, and we found a main effect of Language [*F*(1, 29) = 7.488, *p* = 0.01, *η*^*2*^ = 0.205] and a significant interaction of Language and Gaze [*F*(2, 58) = 13.418, *p* = 0.0001, *η*^*2*^ = 0.316] in the comparison of Normal Speech and Backward Speech. In the comparison of the Normal Speech and No Speech conditions, however, we found no significant difference, neither for Language or Gaze, nor for their interaction.

Bonferroni-corrected post-hoc pairwise tests revealed that infants looked significantly faster at the object in the Congruent Object-Directed Gaze/Normal Speech condition than in the Congruent Object-Directed Gaze/Backward Speech condition [*t*(29) = −5.064, *p* = 0.0001] or in the Congruent Object-Directed Gaze/No Speech condition [*t*(29) = −3.903, *p* = 0.001]. Within the Normal Speech condition latency of orienting towards the object in the Congruent Object-Directed Gaze condition was also significantly shorter compared to the Infant-Directed Gaze condition [*t*(29) = −5.092, *p* = 0.0001], but only close to significant in the Incongruent Object-Directed Gaze condition [*t*(29) = −2.456, *p* = 0.02].

**Figure 3:**
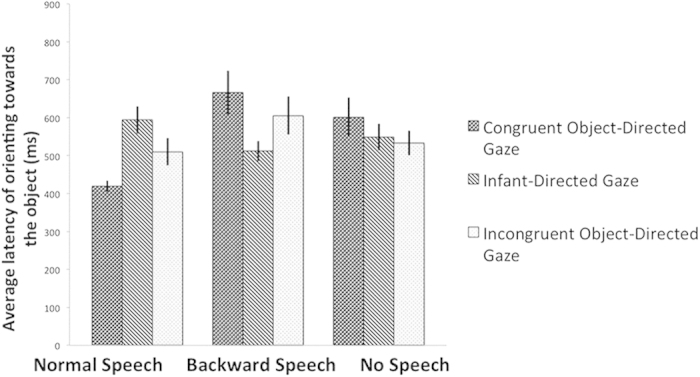
Results of Experiment 2. Latency of fixation towards the visual object. Bars represent the average latency of thirty infants’ orienting towards the visual object in the nine conditions of Speech type x Gaze type. Error bars indicate SEM.

We found no significant difference between the Backward Speech and the No Speech condition in the Congruent Object-Directed Gaze trials. Similarly, we found no difference between the Backward Speech and No Speech conditions in the Infant-Directed Gaze trials either. These results thus replicated the results of the first experiment, by showing that infants’ orientation towards the visual object is the fastest when they hear normal speech and follow the object-directed gaze of the speaker. Furthermore, the results of the second experiment also gave evidence that the object-directed gaze of the speaker is helpful to find the referent of the speech only if it is preceded by speech (Fig. 3). It is important to note that while the general pattern of the results of Experiment 1 was replicated, we found an overall increase of performance in Experiment 2. We assume that this increase is due to the fact that in Experiment 2 we enlarged the size of the face, thus infants found the experiment overall more attractive.

The fact that we did not find a main effect of Language in the comparison of the Normal Speech and No Speech conditions, including all Gaze conditions, rules out the possibility that the demonstrated effect would be a result of either a general increase of arousal in the Normal Speech condition, or a decrease of attention in the silent control condition. However, in order to provide further supporting evidence to this claim, we decided to analyze infants’ pupil dilation during the different auditory conditions, as an indicator or their general attentiveness.

**Figure 4:**
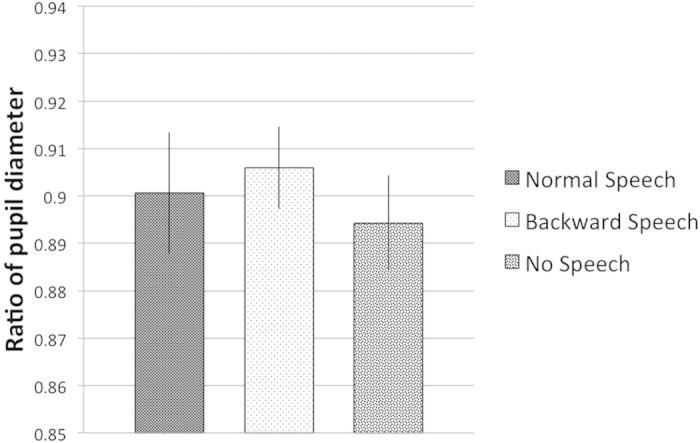
Results of Experiment 2. Changes of pupil diameter during the speech period. Bars represent the average ratio of infants’ pupil size during the period of the presentation of the female speaker in the different auditory (Speech, Backward Speech and No Speech) conditions. Error bars indicate SEM.

**Figure 5:**
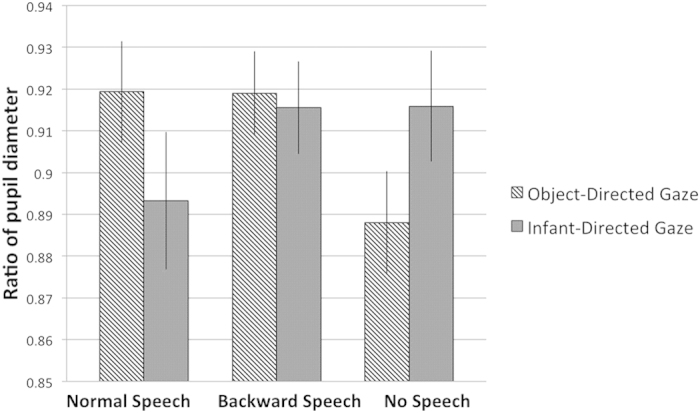
Results of Experiment 2. Changes of pupil diameter during the speech and the gazing period. Bars represent the average ratio of infants’ pupil size during the entire period of the presentation of the female speaker in the different auditory (Speech, Backward Speech and No Speech) and the further Gaze (Object-Directed and Infant-Directed) conditions. Error bars indicate SEM.

Pupil diameter is not only a function of the luminance reaching the eyes, but also reflects psychological processes such as attention, arousal and cognitive load^32^,^33^,^34^ (Beatty, 1982; Karatekin, 2004; Porter, Troscianko, & Gilchrist, 2007), as it has been reported in numerous studies with adult subjects in the past. More recently, it has also been demonstrated that pupil diameter can be informative about psychological processes also in the case of in infants. For example, changes in pupil size have been registered in studies of object-identity violation^35^, object-permanence tasks^36^ or in response to another infant’s distress^37^. Thus, the aim of the analysis of pupil size changes in the present experiment was to see whether the different time latencies infants showed in the behavioural task would be due to an of increase of arousal and general attention enhancement during the presentation of speech. As there is extensive evidence showing that infants have a distinguished preference towards speech stimuli, compared to other, non-speech auditory stimuli, it is especially important to clarify whether the shorter latency in finding the visual objects is due to a generally more ‘alerted’ state that speech would induce in infants, or it is indeed due to a specific effect, reflecting their expectation to find a visual referent of speech. Thus, to answer this question we performed a pupil diameter analysis during the presentation of the female speaker in the different conditions. First, we analysed the period of the different auditory conditions (Speech, Backward Speech and No Speech), in order to see whether infants’ general attentiveness would have changed due to the different acoustic or silent cues. This analysis did not yield any significant differences between conditions. In fact, infants’ average pupil size was very similar in all the three conditions (Fig. 4). Next, we wanted to see whether the combined effect of the auditory conditions and gaze-cueing would result changes of the pupil size. To this end, we analysed changes of pupil diameter during the entire period of the presentation of both the acoustic cue (Speech, Backward Speech and No Speech) and the gaze (Object-directed vs. Infant-directed) (Fig. 5). A a 3 × 3 analysis of variance (ANOVA) with Language (Normal Speech vs. Backward Speech vs. No Speech) and Gaze (Object-Directed vs. Infant-Directed) revealed no main effect of Language or Gaze, but a significant interaction between the two factors [*F*(2, 42) = 7.710, *p* = 0.001, *η*^*2*^ = 0.269]. We found the same interaction when we directly compared the Speech condition with the Backward Speech [*F*(1, 24) = 5.771, *p* = 0.024, *η*^*2*^ = 0.194], and also in the comparison of the Speech and No Speech condition [*F*(1, 21) = 11.825, *p* = 0.002, *η*^*2*^ = 0.360]. In the comparison of the Backward Speech and No Speech condition, however, due to an overall increased pupil size in the Backward Speech condition we found a significant main effect of Language [*F*(1, 23) = 6.629, *p* = 0.017, *η*^*2*^ = 0.224], and only a marginally significant interaction of Language and Gaze [*F*(1, 23) = 4.336, *p* = 0.049, *η*^*2*^ = 0.159]. Further Bonferroni-corrected pairwise comparisons also revealed that pupil size reacted differently to the Object-directed gaze vs. Infant-directed gaze of the speaker only when gazing was preceded by Speech [*t*(24) = 2.831, *p* = 0.009], but not when it was preceded by Backward Speech [*t*(26) = 0.491, *p* = 0.628], or by No Speech [*t*(23) = −2.086, *p* = 0.048]. Thus, while these results clearly exclude the possibility that either normal speech would generally increase infants’ arousal, or that the backward speech and the silent movies would distract their attention, they give further evidence that infants’ searching behaviour for a potential referent is facilitated by the gaze-cueing of the speaker only when the cue appears in the context of speech.

## Discussion

Confirming our hypothesis, the results of Experiment 1 and 2 showed that infants at the age of 4-months are ready to look for potential referents when they are presented with a combination of speech and a referential gaze of the speaker. In both experiments, the latency of orienting towards the object was the shortest when infants heard normal speech, along with an object-directed gaze of the speaker, which was congruent with the direction of the object.

A further intriguing finding of our second experiment is the role of eye-gaze in eliciting referential expectation. While infants in the normal speech condition were the slowest to orient towards an appearing object when the speaker was gazing at them, we found the opposite pattern in the backward speech condition. When infants were exposed to the combination of backward speech and an infant-directed gaze of the speaker, they oriented faster to the object, compared to when the speaker was gazing away from them, even though this effect only reflected a trend and was not significant. Furthermore, when infants saw the speaker moving her lips without producing any sound, eye-gaze did not have any modulatory effect on their orienting towards the objects. What could be the unified explanation for this effect and the faster look in the normal speech condition? Eye-gaze following is a well studied subject of social cognition and cognitive development, and it has been proposed that by directing infants’ attention it can facilitate language acquisition^38^. Studies gave evidence that 8 and 12-month-old infants interpret eye-gaze as a communicative act, and that gazing can elicit referential expectation in the direction of the gaze^39^, but only if the object-directed gaze is preceded by an infant-directed gaze^40^. Furthermore, even newborns can distinguish between eye-gazes that are either directed toward them or averted from them^41^ and at the age of 4-months their cortical processes are enhanced when they perceive a direct eye-gaze, as opposed to when they see an averted gaze^42^,^43^. Thus, already around birth, eye-gaze seems to be a powerful cue in evoking and directing infants’ attention. Based on these findings, could our results be interpreted simply as an effect of the different types of eye-gazes infants perceived independently from the auditory stimuli they heard? Could it be that the different speech conditions merely provided a distracting context? This seems unlikely, since in this case, the latency results in Experiment 2 should show a similar pattern in all the auditory conditions. But the fact that infants reacted differently to the eye-gaze in the three different auditory conditions, more specifically that they were facilitated by the object-directed gaze in the Normal Speech condition, but slowed down in the Backward Speech condition while in the No Speech condition the gaze had no effect at all, means that eye-gaze type and speech type must have a combined effect on their behavior.

Similar to previous proposals^43^, we suggest that during the perception of a direct eye-gaze, infants can recognize the communicative intention, even before they could assess the content of these intentions. Eye-gaze thus is able to establish a communicative context, which can direct the attention of the infant. However, we also suggest that while an infant-directed gaze acts as a communicative cue signaling that the infant was addressed by someone, additional cues are required to elicit the referential expectation of the infant (i.e. to understand that the speaker is talking *about* something). Following this, we propose that when the infant hears speech (without being able to actually understand the content of speech) and observes a person directly gazing at her/him (like in the Infant-directed gaze condition in our experiment), s/he will understand the communicative intention of the speaker (i.e. that s/he was addressed by the speaker), but s/he will still have to wait for additional referential cues to make an inference that the speaker is actually talking *about* something. This additional cue arrives when the direct eye contact is broken: the very moment when the speaker averts her gaze to a new direction, the infant will infer that some new and relevant information is being presented to her via the speech signals, and, as a consequence will be ready to seek this information. Specifically in our experiment, when infants heard normal speech and saw the speaker directly gazing at them, they were simply waiting for a second signal to establish a referential expectation and to start to look for potential referents of the speech. In contrast, in the object-directed gaze conditions, their referential expectation was already evoked when the speaker averted her gaze, and as a result, infants were immediately ready to look for a referent. Accordingly, even when the object-directed gaze of the speaker was incongruent with the appearance of the object, infants were still faster looking at the object, compared to when they were waiting for additional cues of the speaker in the infant-directed gaze condition. Thus, we propose that infants are able to interpret speech in different ways (i.e. either as ostensive-communicative, when they were only addressed by the speaker, or as referential, when the speaker is already talking about something), and in order to choose a certain interpretation, additional cues are required. These cues could involve the eye-gaze of the speaker, or other types of referential cues as well (e.g. pointing^44^).

Within the same communicative-referential context hypothesis, we could only speculate why infants were marginally slower in finding the objects when the backward speaking person also provided an averted referential gaze, compared to when the speaker was gazing at them. From previous studies we know that backward speech cannot be processed similarly to any natural languages^3^,^30^,^31^, thus it cannot establish any communicative-referential context. Moreover, since infants are expecting human faces to produce human sounds^45^, when they perceive backward speech, they likely get puzzled by the strange vocalization of the human face. Thus, it is difficult to make any precise predictions about the effect of the combination of backward speech and eye-gaze of the speaker in eliciting referential expectation. The marginal slowdown might be because infants got confused by perceiving a referential cue from a person, who was at the same time producing non-human sounds, and they were waiting for additional referential cues (e.g. normal speech), to eliminate their confusion, by attaining coherent cues of the speaker.

A further question is why in the No Speech condition eye-gaze did not have any modulatory effect on infants’ looking time latency. This result seems to conflict with studies showing that both newborns and infants from a very early age tend to follow the direction of eye-gaze movements^35^. However, while typical studies on gaze following do not involve speech as an additional variable, in our experiments infants always saw a person who was either talking or silently moving the lips. Hence, infants in the No Speech condition were not simply influenced by the eye-gaze of the actor, but also by the lack of speech, which was even more salient, since the actor was still moving her lips, but without producing any sounds, and this might have had a different effect on them. Thus, we posit that our study cannot be directly compared to those investigating the effect of eye-gaze cuing, because in our experiments we used a combination of speech vs. lack of speech, which could have resulted a different effect from previous studies.

One may argue that the perceived shorter latency, rather than reflecting infants’ referential expectation about speech, could be simply either a result of an increase of arousal level, due to the speech condition, or a decrease of infants’ attention, due to the ‘strangeness’ of the backward and silent conditions. However, both the results of the behavioral experiment and the further pupil diameter analysis of Experiment 2 seemed to rule out this possibility. The fact that in Experiment 2 we found significant difference of the latency between the Normal Speech and the other two auditory conditions only in the Congruent Object-Directed Gaze condition, but not in the two other Gaze conditions, excludes the possibility that the demonstrated effect would be due to an overall attention enhancement in the Normal Speech condition. In fact, if speech would elicit a general increase of arousal, we should find an overall increase of performance in all gaze conditions, but our results did not confirm this. Another possibility is that rather than speech would facilitate infants’ searching behavior, it might be that the two other conditions slowed them down, since both of them might serve as unnatural stimuli for the infants. If this was the case, then we should find an overall worse performance in the Backward Speech and No Speech conditions, compared to the Normal Speech Condition. However, we only found a difference in the comparison of the Normal Speech and Backward Speech condition, but not between the Normal Speech and the No Speech condition, when we calculated the overall performance in the different Gaze conditions. Thus, we can exclude the possibility that our control conditions would have distracted subjects’ performance, resulting an overall decrease of latency of fixation. Furthermore, additional data about changes of pupil diameter also confirmed these results. While during the period of the three auditory conditions we found similar values of average pupil size, reflecting the same arousal level in the different conditions, in the period of the averted gazing infants’ attention got modulated only if previously they were listening to the normal speech.

Hence, we propose that the only explanation for both the shorter time taken to orient toward the object and for the changes of pupil size is that they reflect infants’ readiness to find a potential referent of speech in the environment of the speaker, which supports the idea that infants at this age already show an important precursor in understanding the referentiality of language.

Our starting hypothesis was that understanding the referential nature of language might be a human-specific predisposition of infants. By testing two groups of young infants, we gave evidence that when young infants are exposed to a combination of speech and an object-directed gaze of the speaker, this will elicit referential expectation and bias them to start to look for potential referents of speech. However, since in our study we tested 4-month-old infants, we cannot exclude the possibility that infants at this age are also influenced by their experiences of the visual world, and that the referential expectation they express is a result of what they learned during their experience up to that point. In order to support a statement regarding the innateness of referential expectation about speech, one would need to show the same effect with neonates, who do not have yet much exposure to the visual world.

## Conclusion

Our results highlight the fact that speech can be interpreted in different ways (i.e. solely ostensive-communicative or also referential) very early in the development, but that additional cues are required to choose between these possible interpretations. When a speaker only provides ostensive cues along with speech (i.e. a direct-eye gaze), the interpretation is restricted to the fact that s/he wants to elicit the attention of the infant. However, when additional referential cues are also provided (i.e. an averted, thus possibly object-directed gaze of the speaker), this will establish a referential interpretation of speech, and infants will be ready to seek for potential referents. We found that infants at a very early age are already able to express signs of these different interpretations when they are exposed to speech and a following eye-gaze of the speaker.

Our findings shed a new light on the early learning mechanisms of infants. The fact that already at this early age infants show a precursor in understanding that language is a possible tool to convey messages and transfer knowledge means that they are also ready to learn about the world via their conspecifics. By being predisposed to get information from their social partners, infants can selectively attend to certain stimuli in their environment. Thus, by talking to infants, even if they do not understand the meanings of the words yet, one can effectively draw their attention towards distinct elements of their surroundings and, as a consequence, shape their learning processes from a very early age on.

An intriguing question is whether this early precursor in considering spoken words, as abstract symbols is restricted only to linguistic stimuli, or it reflects a more general understanding of the existence of symbols, the fact that, independently from modality, certain stimuli (gestures, sounds, pictures) can represent and stand for other entities in the world. Indeed, there is some evidence that while young infants initially have a tendency to accept a wide range of entities as potential symbols, later they start to develop a unique preference towards linguistic stimuli^46^. Whether or not this means that they also have an innate understanding that regardless of modality, any types of entities could potentially be understood as representations for other entities, is a subject of future research.

## Materials and Methods

### Participants

Infants ranged in age from 3 months and 14 days to 4 months and 12 days, and were from Italian-speaking families. In Experiment 1 we tested 48 infants, from which 10 were excluded due to uncompleted trials, 6 because of fussiness and 2 because of experimental error. In Experiment 2 we tested 37 infants, from which we excluded 5 due to missing trials and 2 because of fussiness. Infants’ mean age was 4 months, 05 days in Experiment 1, and 4 months, 11 days in Experiment 2.

Our study was approved by our institutional review board, the Bioethics Committee of the International School for Advanced Studies. All of our experiments followed the guidelines of this committee and all of our protocols were approved by the committee. After being informed about the procedure, the parents of all participants provided written consent.

### Stimuli

Infants were presented with videos of a female face and a following object. In Experiment 1 during Normal Speech trials the female face first looked at the infant for 1 second, and said “Ciao bambina! Guarda! (Hi Baby! Look!) and then a pseudo word ”A gabato/lumipa/falasi!”, and finally she looked either to the left, or to the right side of the screen for 2 seconds. Then the face disappeared and a colorful toy immediately appeared for 2 seconds. In the Backward Speech trials we reversed the movies, so the speaker produced the same sentences in backward speech, then looked to one side, where a subsequent object appeared. In the No Speech Condition everything was the same, except that the sound was deleted from the movies. For the objects we used pictures of 8 colorful toys with a size of approximately 25 × 25 cm. In Experiment 1 infants saw 24 movies, 8 in each condition. In Experiment 2 along with the previous stimuli types we also included trials when the direction of the eye-gaze of the speaker was incongruent with the appearance of the subsequent object, and trials what ended by an infant-directed gaze of the speaker (2 seconds). In Experiment 2 infants saw 27 movies, 3 in each condition (see Supplementary materials).

### Apparatus and Procedure

Infants sat on a parent’s lap 80 cm from a 17-in. LCD screen in a soundproof booth. Parents were wearing eye-glasses covered by stickers to prevent them from being able to see the videos and influence the infants’ behaviour. Stimuli were played by a Psyscope B70 software that was controlled from outside by the experimenter. The sound was played from a loudspeaker located behind the screen. Infants were also videotaped during the experiment. Once the experimenter started a trial, the fixation attractor disappeared on the screen for 2 seconds and then a movie started. A Tobii T-120 Eye-tracker system recorded infants’ eye-movements during the experiment. Data was recorded at a sampling rate of 60 Hertz. Before starting the recording, we performed a 5-point calibration. Attractors were presented one after the other in each corner and in the center of the screen. For each attractor, we waited for the position of the gaze to stabilize, before presenting the next one. The difference between the estimated gaze position and the position of the attractor where infants were gazing at was used for the calibration. If calibration was not successful it was repeated. No infant was excluded due to failure of calibration.

To assess for temporal drift, for each trial the difference of the timestamp between consecutive samples was obtained. Given that the recording rate was at 60 Herz, the time difference between two samples had to be 16.6 msec. We found no significant deviation from this value.

### Data analysis of latency of fixation at the visual object

We defined three windows of interest for the analysis of the infant eye gaze, by dividing the screen to a center, a right and a left part. Only those trials, which contained at least a 3 sec long fixation of the infant towards the center while the face was displayed, before the appearance of the object, were included in the analysis. Trials not meeting these criteria were excluded due to possible ignorance of the face of the actor. Furthermore, in Experiment 1 infants had to complete at least 4 trials in each condition, whereas in Experiment 2 they had to complete at least 2 trials in each condition, otherwise they were excluded from the data analysis. As dependent variable for the main analysis, we considered the latency of the first fixation on the object in each trial. Latency was calculated as the time to the first fixation on the region of interest where the object was presented, after object presentation. We defined a fixation as a block of at least 5 consecutive samples (83.33 ms) inside a ROI.

### Data analysis of pupil diameter changes

To calculate changes of the pupil diameter, throughout the duration of the experiment, we registered both eyes’ pupil diameter in millimetres. We removed artifact caused by head movement and saccades from the continuous pupil diameter data by eliminating extreme values (lower and top percentile) for each eye independently, after which we averaged across both eyes to further reduce noise. Next, we proceeded to extract, for each trial, the pupil diameter data on two time windows of interest. The first window spanned 4 seconds and was located during the presentation of the fixation cross at the beginning of each trial (Fixation Cross period). The purpose of this window was to be used as a baseline. Our second window of interest spanned a mean of 4.5 seconds and was located during period in which the female speaker produced the different auditory conditions (Auditory Condition period). From this point, we only included in the analysis, trials for which we registered a minimum of 50% of valid samples during the first window of interest (fixation cross) and a minimum of 70% of valid samples during the second windows on interest. Through this procedure, 21.2% of the total amount of trials was rejected. For each trial, we calculated the mean inside each time window of interest. In order to normalize the data, pupil diameter for the second time window was expressed in terms of ratio of with respect to the first window. We also performed a second analysis, identical to this one, but taking a time window that spanning the entire presentation of the female speaker, including the gazing. The mean duration of this window was 7.5 seconds and 19.55% of the total amount of trials was rejected.

## Additional Information

**How to cite this article**: Marno, H. *et al.* Can you see what I am talking about? Human speech triggers referential expectation in four-month-old infants. *Sci. Rep.* **5**, 13594; doi: 10.1038/srep13594 (2015).

## Supplementary Material

Supplementary Information

Supplementary Movie 1

Supplementary Movie 2

Supplementary Movie 3
