# Supplementary material for: Can you see what I am talking about? Human speech triggers referential expectation in four-month-old infants
Source: Sci Rep. 2015 Sep 1;5:13594. doi: 10.1038/srep13594 (PMC4555167; doi:10.1038/srep13594)
Supplement: Supplementary Information [file srep13594-s1.pdf]

Can you see what I am talking about? Human speech triggers referential expectation in four-month-old infants

Hanna Marno, Teresa Farroni, Yamil Vidal Dos Santos, Milad Ekramnia, Marina Nespor and Jacques Mehler

Supplementary Material File Names

S1. Example for the Normal Speech condition.mov

S2. Example for the Backward Speech condition.mov

S3. Example for the Silent condition.mov

Supplementary Material Legends

SI 1. An example for the Normal Speech Condition with an object-directed eye-gaze to the left side.

SI 2. An example for the Backward Speech Condition with an object-directed eye-gaze to the right side.

SI 3. An example for the Silent Condition with an object-directed eye-gaze to the left side.
